# NUMBER OF FRIENDS AND THE RISK OF DEVELOPING DIABETES: A REPLICATION USING NSHAP AND HRS

**DOI:** 10.1093/geroni/igz038.1627

**Published:** 2019-11-08

**Authors:** Louise Hawkley, Phil Schumm, Elbert Huang, Martha McClintock

**Affiliations:** 1 NORC, Chicago, Illinois, United States; 2 Biostat Consulting Lab, University of Chicago, Chicago, Illinois, United States; 3 General Internal Medicine, University of Chicago, Chicago, Illinois, United States; 4 Institute for Mind & Biology, University of Chicago, Chicago, Illinois, United States

## Abstract

The epidemic increase in diabetes prevalence (primarily type 2) is a public health crisis. We hypothesized that the rates of movement among diabetic states depend in part on one’s social relationships and environment. Using population-based samples from both NSHAP and HRS, collected in 2005–15, we found that having more friends was associated with a lower risk for acquiring diabetes over the next 4-5 years. As an independent replication, separate logistic models for NSHAP and HRS data yielded similar odds-ratios for the protective effect of having friends (OR = 0.82 and 0.92 respectively), adjusting for gender, age, race/ethnicity, and BMI. This effect was concentrated entirely between 0–4 friends; differences in the number of friends above 4 were not associated with differences in diabetes risk.

